# Safety and Efficacy of Novel Morphology Classification-Guided Mitral Valve Transcatheter Edge-to-Edge Repair for Patients With Commissural Degenerative Mitral Regurgitation: Design and Rationale of the TEER-CD Trial

**DOI:** 10.31083/RCM39373

**Published:** 2025-12-19

**Authors:** Yang Li, Xu-Nan Guo, Yihang Wu, Yutong Ke, Xianbao Liu, Shih-Hsien Sung, Junjie Zhang, Tao Chen, Zuyi Yuan, Guosheng Fu, Bin Wang, Yangxin Chen, Xiaoping Peng, Xiaodong Zhuang, Yining Yang, Saibal Kar, Yat-Yin Lam, Guangyuan Song

**Affiliations:** ^1^Interventional Center of Valvular Heart Disease, Beijing Anzhen Hospital, Capital Medical University, 100029 Beijing, China; ^2^Echocardiography Department, Beijing Anzhen Hospital, Capital Medical University, 100029 Beijing, China; ^3^Department of Cardiology, The Second Affiliated Hospital, Zhejiang University School of Medicine, 310009 Hangzhou, Zhejiang, China; ^4^Division of Cardiology, Department of Medicine, Taipei Veterans General Hospital, 407219 Taipei, Taiwan, China; ^5^Department of Cardiology, Nanjing First Hospital, 210029 Nanjing, Jiangsu, China; ^6^Department of Cardiology, The Second Affiliated Hospital of Harbin Medical University, 150086 Harbin, Heilongjiang, China; ^7^Department of Cardiology, The First Affiliated Hospital of Xi'an JiaoTong University, 710063 Xi'an, Shaanxi, China; ^8^Department of Cardiology, SIR RUN RUN SHAW Hospital, Zhejiang University School of Medicine, 310016 Hangzhou, Zhejiang, China; ^9^Department of Emergency, Xiamen Cardiovascular Hospital, Xiamen University, 361016 Xiamen, Fujian, China; ^10^Department of Cardiology, Sun Yat-Sen Memorial Hospital, Sun Yat-Sen University, 510123 Guangzhou, Guangdong, China; ^11^Department of Cardiology, The First Affiliated Hospital of Nanchang University, 330209 Nanchang, Jiangxi, China; ^12^Department of Cardiology, The First Affiliated Hospital, Sun Yat-Sen University, 510060 Guangzhou, Guangdong, China; ^13^Department of Cardiology, People's Hospital of Xinjiang Uygur Antonomous Region, 830094 Urumqi, Xinjiang, China; ^14^Department of Cardiology, Los Robles Regional Medical Center, Thousand Oaks, CA 91360, USA; ^15^Division of Cardiology, Department of Medicine and Therapeutics, Asian Heart Disease Center, Canossa Hospital, The Chinese University of Hong Kong, Hong Kong, China

**Keywords:** degenerative mitral regurgitation, transcatheter edge-to-edge repair, MitraClip, morphological classification, valvular heart disease

## Abstract

**Background:**

Mitral commissural prolapse or flail, characterized by intricate and diverse anatomical features, poses a significant challenge in mitral transcatheter edge-to-edge repair (M-TEER). Previous studies have largely focused on central mitral regurgitation with favorable valve anatomy or a general broad spectrum of complex mitral regurgitation. However, no established approach is currently available for M-TEER in commissural degenerative mitral regurgitation (DMR).

**Methods:**

Therefore, this study aimed to evaluate the efficacy and safety of a novel morphology classification-guided M-TEER strategy for treating commissural DMR using the MitraClip system. This prospective, multicenter, single-arm, objective performance criteria study involved 12 experienced centers in Asia, primarily located in China. Patients with symptomatic moderate-to-severe (3+) and severe (4+) native DMR and commissural involvement were stratified into three morphological categories based on an echocardiographic core laboratory analysis, and tailored M-TEER strategies were proposed. The primary endpoint is the proportion of patients achieving a mitral regurgitation (MR) grade of ≤1+ without repeat mitral intervention at one-year follow-up. Clinical, echocardiographic, functional, and quality-of-life outcomes were assessed over one year.

**Results:**

Based on statistical power calculations, a total of 148 patients are required to achieve adequate power to test the primary efficacy hypothesis, accounting for an estimated 10% attrition rate at 12 months.

**Conclusions:**

The morphology classification system enhances M-TEER for commissural DMR by addressing the unique challenges of this approach, enabling tailored interventions that optimize procedural success and patient outcomes.

**Clinical Trial Registration:**

ChiCTR2400090258, https://www.chictr.org.cn/showproj.html?proj=239191.

## 1. Introduction

Mitral regurgitation (MR) is a prevalent valvular heart condition, with degenerative MR (DMR) affecting at least 24 million people worldwide [1]. Within the spectrum of DMR, commissural lesions represent a significant subset, characterized by prolapse or flail involving the mitral valve commissures. These lesions, while less common than central scallop prolapse, pose unique challenges due to their anatomical location and the complex interplay of the mitral valve apparatus. However, epidemiological studies suggest that commissural DMR is often under-recognized and can be associated with more advanced disease at the time of diagnosis, partly due to the subtle and variable presentation on standard echocardiographic evaluation [2].

Mitral Transcatheter Edge-to-Edge Repair (M-TEER) therapy has revolutionized the treatment of MR by offering a minimally invasive alternative to surgery [3, 4]. However, the treatment of commissural lesions remains particularly challenging. Unique anatomical factors—such as the difficulty in accessing and visualizing the commissural regions—combined with the technical demands of the procedure and devicerelated challenges, including the need for transseptal puncture (transfemoral MTEER), the long access route, the risk of clip entanglement with chordae tendineae, and the limited grasping range of current devices, contribute to the higher rates of residual or recurrent MR observed in this subgroup [5]. Notably, prior literature has reported that, among patients with commissural DMR treated with M-TEER, only 45% achieved MR ≤1+ at discharge and 33.3% at 3-year follow-up [6], underscoring the procedural complexity and suboptimal long-term durability in this challenging subset. Additionally, the lack of standardized classification systems and tailored procedural strategies for commissural DMR has hindered the optimization of M-TEER outcomes. Besides, some investigators have proposed an adjunctive technique involving MitraClip implantation followed by the placement of an Amplatzer Vascular Plug (AVP) II between the commissure and the MitraClip to address residual regurgitation in commissural DMR [7]. While technically feasible, this approach has been associated with a relatively high incidence of post-procedural hemolysis [8], which has limited its widespread adoption. Furthermore, patients with commissural lesions are frequently excluded from clinical trials or are represented by a small proportion of the study population, which limits the generalizability of current treatment guidelines.

Considering the lack of evidence on M-TEER therapy for commissural DMR, we designed a prospective, multicenter, single-arm clinical investigation. The Mitral Valve Transcatheter Edge-to-Edge Repair for Patients with Commissural Degenerative Mitral Regurgitation (TEER-CD) study aims to address these gaps by focusing on the development and validation of a novel morphological classification system specifically designed for commissural DMR. This system is designed to standardize patient selection and procedural techniques, thereby potentially improving the safety and efficacy of M-TEER in this challenging patient population. By doing so, the study seeks to broaden the applicability of M-TEER and provide a much-needed evidence base for the treatment of commissural DMR.

## 2. Materials and Methods

### 2.1 Study Design and Objectives

TEER-CD (registered at https://www.chictr.org.cn/, identifier ChiCTR2400090258) is a prospective, multicenter, single-arm, objective performance criteria clinical trial designed to rigorously evaluate the safety and efficacy of a novel morphological classification-guided M-TEER approach for patients afflicted with commissural DMR. This study may represent a further advancement in the field of interventional cardiology, focusing on a patient population that has historically been challenging to treat with conventional M-TEER methods. The study aims to demonstrate that this novel strategy can achieve safety and effectiveness comparable to that observed for central DMR using current standard of care M-TEER approaches.

The primary objective of this trial is to assess the safety and effectiveness of the MitraClip system (Abbott, Abbott Park, IL, USA) in patients with symptomatic, moderate-to-severe (3+) or severe (4+) native commissural DMR. The trial is designed to demonstrate that outcomes with the novel morphological classificationguided approach for commissural DMR are equivalent or similar to those achieved for central DMR, consistent with American College of Cardiology/American Heart Association (ACC/AHA) and European Society of Cardiology (ESC) guideline recommendations [9, 10].

The TEER-CD trial design and endpoints were developed by the study investigators and steering committee in accordance with the definitions outlined by the Mitral Valve Academic Research Consortium (MVARC) [11]. The TEER-CD trial is jointly funded by Beijing Anzhen Hospital and Abbott Medical. Participating centers have obtained approval from an institutional ethics committee. Informed consent forms are provided to the subjects in the trial.

### 2.2 Imaging Protocol

#### Determination of Commissural Degenerative Mitral Regurgitation

MR associated with commissural degenerative disease is characterized by specific anatomical abnormalities primarily involving the mitral valve commissures. The commissures, where the anterior and posterior mitral leaflets meet, are critical points of coaptation during systole and are prone to degenerative changes [2, 12].

The anatomical determination of mitral commissures is essential for accurate diagnosis and treatment planning. The mitral valve is separated by two commissures: the anterolateral commissure (AC) and the posteromedial commissure (PC). These commissures are identified by the characteristic chordae tendineae that merge, creating a fan-like appearance, and are essential for leaflet coaptation [2]. In clinical practice, the cleavage segment that separates the anterior leaflet from the posterior leaflet is used as an imaging approximation for the true anatomic commissures, especially when individual chordae tendineae attachments are difficult to discern via transesophageal echocardiography (TEE).

**Fig. 1.**
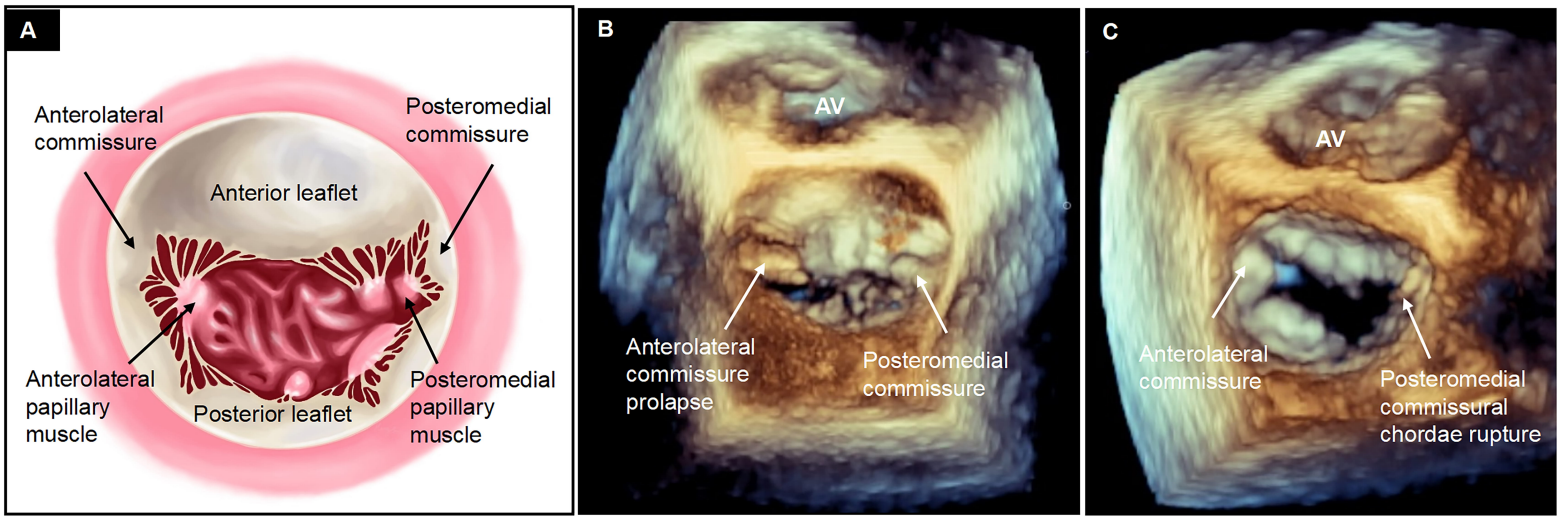
**Definition of commissural degenerative mitral regurgitation**. (A) Commissures, where anterior and posterior mitral leaflets converge during systole, typically form a Y-shaped structure by joining three segments (e.g., A1-anterolateral commissure-P1 or A3-posteromedial commissure-P3), distinguished by fanlike chordae tendineae stemming from anterolateral and posteromedial papillary muscles. Given the difficulty in distinguishing individual chordae on transesophageal echocardiography, the cleavage segment that separates the anterior leaflet from the posterior leaflet is used as imaging for anterolateral and posteromedial commissure approximation of true anatomic commissures. (B) The protrusion of the commissural segment into the left atrium during systole or (C) the rupture of commissural chordae tendineae characterizes commissural prolapse or flail [13]. AV, aortic valve.

The morphological abnormalities defining commissural DMR include prolapse or flail of the commissural segments. The diagnosis is confirmed by the observation of abnormal protrusion of the commissural segment into the left atrium during systole due to elongation or rupture of the commissural chordae tendineae. This is evident in both two-dimensional (2D) and three-dimensional (3D) echocardiographic imaging, which is crucial for the classification and subsequent procedural strategy for M-TEER (Fig. 1, Ref. [13]).

**Fig. 2.**
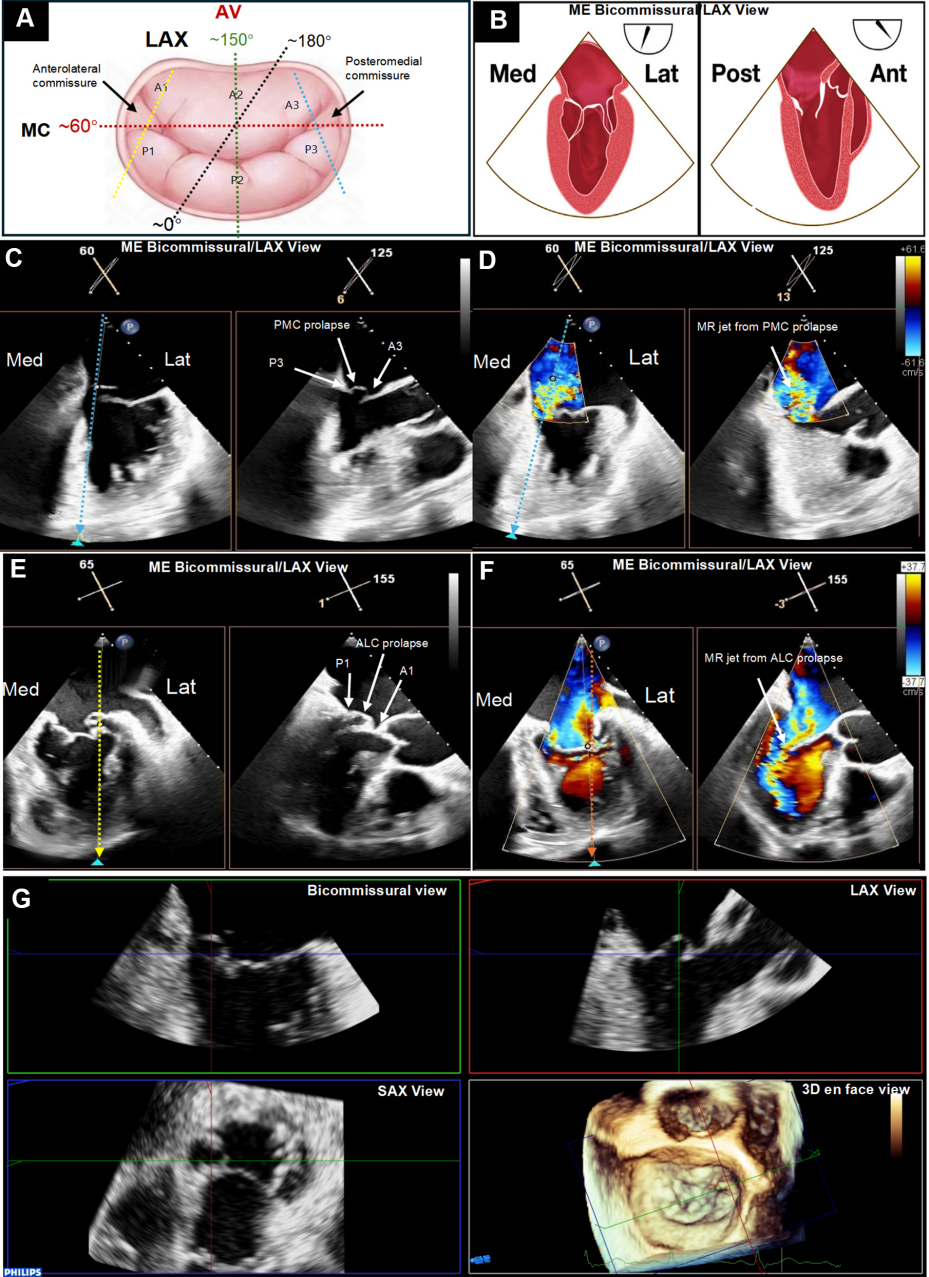
**Imaging protocol for mitral valve commissural disease**. (A) Illustration of the mitral valve (MV), surgeon’s view in the “clock-face plane” with the aortic valve (AV) at the 12 o’clock. Transesophageal echocardiography is used to identify mitral commissural (bicommisural) view anatomically optimized the MV plane (~50°–70°, the red dash line), and long axis (LAX) view best clarified MV coaptation plane, such as ~120°–150° for central segments (the green dash line), >~150° for lateral segments (the yellow dash line), and <~130° for medial segments (the blue dash line). (B) Simultaneous biplane imaging permits the use of a dual screen to display two real-time two-dimensional images simultaneously. The first (primary) image of the MV commissure view can be used as the reference view, with the second view, LAX view rotated from 0° to 180° from the primary view to sweep the interrogation of the MV coaptation (central, lateral, and medial tilts). (C) Example of biplane imaging with (D) color Doppler simultaneously displays bicommissural view (~50°–70°) and modified LAX view to identify the posteromedial commissural lesions. (E) Example of biplane imaging with (F) color Doppler simultaneously displays bicommissural view (~50°–70°) and modified LAX view to identify anterolateral commissural lesions. (G) Three-dimensional rendered enface view with multiplanar reconstruction technique is used to determine the precise mechanism of mitral regurgitation and morphological characteristics of the MV [13]. 3D, 3-dimensional; AC, anterolateral commissure; Ant, anterior; Lat, lateral; MC, mitral commissure; ME, midesophageal; Med, medial; MR, mitral regurgitation; MV, mitral valve; Post, posterior; SAX, short axis; PC, posteromedial commissure.

A standardized TEE protocol was implemented at each participating site and monitored by the echocardiographic core laboratory (ECL). Initial imaging was conducted at the mid-esophageal level to assess the mitral valve. If this level did not yield high-quality images, imaging at the transgastric level was mandated. The protocol included 2D single-plane and simultaneous multiplane imaging to delineate the pathology of MR. The primary reference view, the mid-esophageal mitral commissural view, was primarily used to evaluate the distribution of the regurgitant jet. The secondary view, the long axis view, was aligned perpendicular to the mitral valve coaptation line to further characterize the regurgitant pathology. 3D TEE enhanced with multiplanar reconstruction provided a detailed visualization of the mitral valve anatomy. Color Doppler imaging was employed across these views to accurately localize the origins of the mitral regurgitant jets (Fig. 2, Ref. [13]) [14]. The severity of MR was assessed by the ECL at baseline and follow-up using a comprehensive analysis of quantitative and semiquantitative criteria according to the American Society of Echocardiography [15].

### 2.3 Morphology Classification–Guided M-TEER Procedure and Strategy for Commissural DMR

**Fig. 3.**
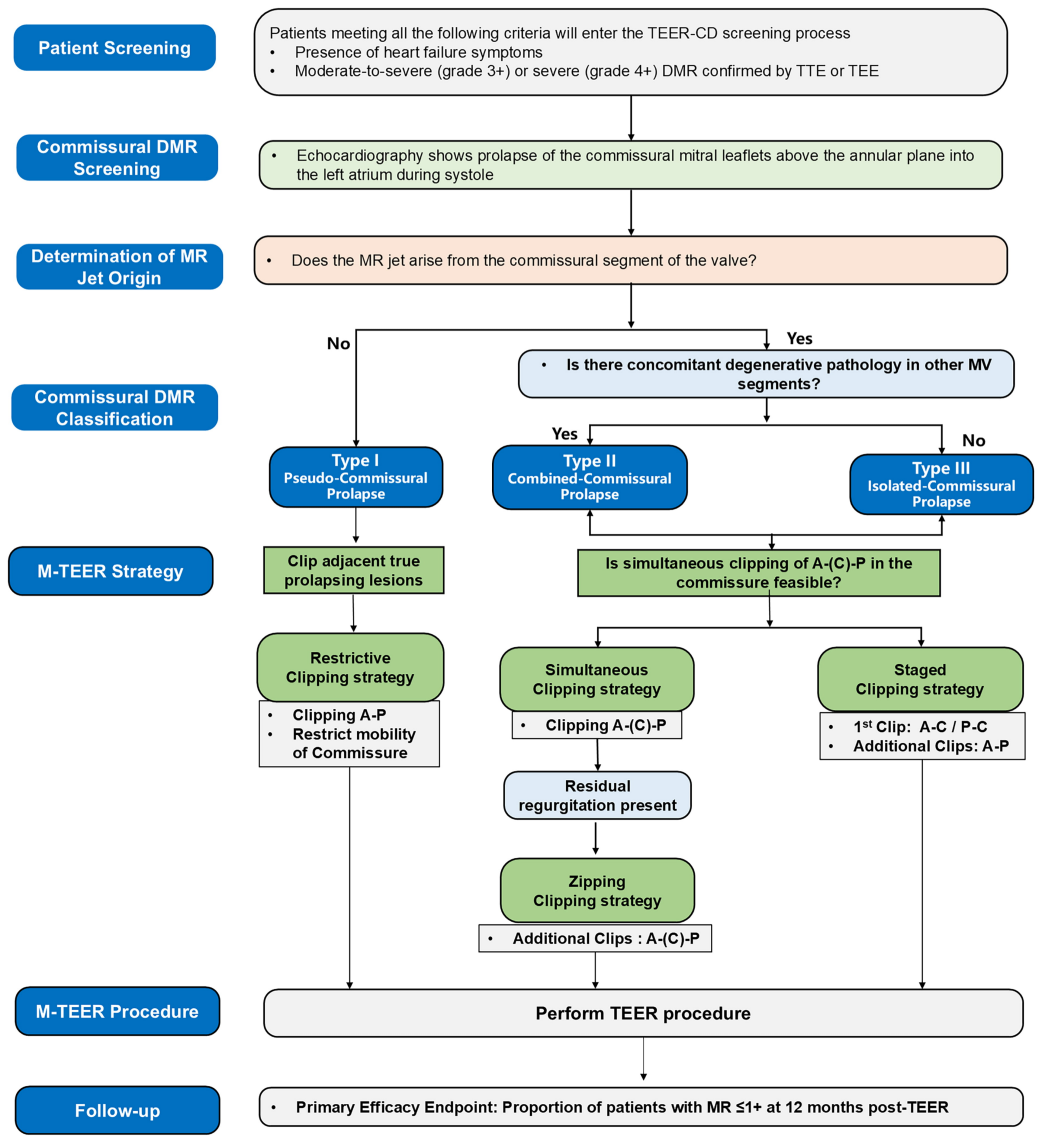
**Flowchart of the novel morphology classification-guided M-TEER strategy for commissural degenerative mitral regurgitation**. A, anterior leaflet; C, commissure leaflet; DMR, degenerative mitral regurgitation; M-TEER, mitral transcatheter edge-to-edge repair; MV, mitral valve; P, posterior leaflet; TEE, transesophageal echocardiography; TEER, transcatheter edge-to-edge repair; TTE, transthoracic echocardiography; MR, mitral regurgitation; TEER-CD, Mitral Valve Transcatheter Edge-to-Edge Repair for Patients with Commissural Degenerative Mitral Regurgitation.

In this study, the morphology classification of commissural DMR and the corresponding classification-guided M-TEER strategy will be determined according to the following steps (Fig. 3). First, determine whether the mitral regurgitant jet originates from the commissural region. If no commissural regurgitant jet is present, the lesion is classified as Type I (pseudo-commissural prolapse). For Type I patients, a restrictive clipping strategy is planned, aiming to clip the true prolapsed lesion adjacent to the involved commissural area while restricting excessive motion of the commissural leaflet. If a commissural regurgitant jet is present, further assess whether degenerative mitral valve lesions are also present in regions other than the commissure. Patients with additional degenerative lesions outside the commissural region are classified as Type II (combined commissural prolapse), whereas those without are classified as Type III (isolated commissural prolapse). For Type II and Type III patients, the next step is to evaluate leaflet length and commissural space to determine whether the three coaptation lines—A-(C)-P—can be grasped simultaneously. If simultaneous grasping is feasible, the simultaneous clipping strategy is applied, clipping the A-(C)-P leaflets together in a single grasp. If, after release of the first clip, significant residual regurgitation persists, additional clips may be implanted to treat the remaining diseased A-(C)-P leaflet segments, forming a zipping clipping strategy. If simultaneous grasping of the A-(C)-P coaptation lines is not possible, a staged clipping strategy is recommended, completing repair of the three coaptation lines in two steps: the first clip is used to grasp the A-C or P-C segments to create a unified tissue bridge, followed by implantation of an additional clip to perform edge-to-edge repair with the adjacent P or A leaflet. The schematic illustration of the procedural strategy is provided in **Supplementary Fig. 1**.

The M-TEER procedure was performed under general anesthesia with strict adherence to ultrasound-guided vascular access, using integrated fluoroscopic, 2D, and 3D-TEE guidance. After induction of anesthesia, a guidewire and catheter were advanced via the femoral vein under ultrasound guidance. The device was delivered through a transeptal puncture, entering the left ventricle via the mitral valve. Optimal transeptal puncture height was targeted at 3.5–4.0  cm for AC pathology and 4.0–4.5  cm for PC pathology. Device positioning and deployment aimed to grasp and approximate the mitral leaflets, thereby reducing regurgitation. Continuous 2D and 3D-TEE monitoring was essential for guiding clip placement, ensuring perpendicular alignment to the coaptation line, and confirming reduction of regurgitation after clip deployment. A 3D enface view of the mitral valve was used to verify device orientation, and adjustments were made in real time based on imaging feedback to optimize procedural outcomes. Each participating center is encouraged to review the morphological classification for each patient and determine the optimal M-TEER strategy, taking into account patientspecific anatomy and the center’s procedural expertise.

### 2.4 Device Description

In this study, we will employ the MitraClip G3 or G4 System (Abbott, Abbott Park, IL, USA), a state-of-the-art transcatheter device for the treatment of significant symptomatic MR 3+ or 4+ grade. This system provides a nuanced selection of clip sizes, meticulously tailored to accommodate diverse patient anatomies. Specifically, the NT (normal length, standard (thin) width) and XT (extended length, standard width) clips feature a compact design with a traditional width of 4 mm and lengths of 9 mm and 12 mm, respectively. The NTW (normal length, wide width) and XTW (extended length, wide width) clips are engineered for broader anatomical needs, with dimensions of 6 mm in width and lengths of 9 mm and 12 mm, respectively. These dimensions facilitate a high degree of procedural customization essential for effective mitral valve repair. Each clip is designed for independent leaflet grasping and is compatible with left atrial pressure monitoring, features that significantly enhance the precision and adaptability of M-TEER for commissural DMR.

### 2.5 Patient Population

Eligible patients have symptomatic (i.e., New York Heart Association [NYHA] functional classification II/III/IV), or asymptomatic heart failure (HF) with left ventricular end-systolic diameter >40 mm; have moderate-to-severe (3+) or severe (4+) commissural DMR as confirmed by the study ECL prior to enrollment; and MR can be reduced to mild or less with the MitraClip device. A complete list of the TEER-CD trial inclusion and exclusion criteria appears in Table 1.

**Table 1.** **Inclusion and exclusion criteria**.

Inclusion criteria (all must be present)
∙ Age ≥18 years old
∙ Moderate-to-severe (3+) or severe (4+) commissural DMR, as determined by an independent ECL using standardized echocardiographic assessments (multiple etiologies are acceptable, but the main mechanism of mitral regurgitation must be degenerative)
∙ Symptomatic HF consistent with NYHA functional class II, III, or IV, or be asymptomatic but with evidence of cardiac dysfunction, such as LVESD ≥40 mm
∙ The presence of atrial septum and mitral valve anatomy that are amenable to percutaneous edge-to-edge repair as confirmed by the ECL and multidisciplinary heart team
∙ Comply with all provisions of this clinical trial and to participate in all necessary postprocedural follow-ups, and to provide a written informed consent form
Exclusion criteria (all must be absent)
∙ DMR not involving the commissural regions will be excluded to focus on the specific anatomical subset addressed by this study
∙ Presence of moderate or greater FMR [defined as central MR of grade 2+ or higher, caused by malcoaptation of the mitral leaflets outside the degenerative commissural mitral regurgitation lesion, restrictive cardiomyopathy, hypertrophic cardiomyopathy, dilated cardiomyopathy, ischemic cardiomyopathy, and infiltrative cardiomyopathy (amyloidosis, hemochromatosis, sarcoidosis, etc.)]
∙ History of mitral valve surgery or transcatheter mitral valve intervention
∙ Concurrent other moderate-to-severe (3+) or severe (4+) valvular disease requiring surgical or transcatheter intervention
∙ Acute cerebrovascular disease within 30 days
∙ Other cardiovascular surgical or interventional treatments within 30 days, such as CABG, PCI, TAVR, transcatheter carotid stenting, etc.
∙ Severe symptomatic carotid artery stenosis (ultrasound examination showing stenosis >70%)
∙ Hemodynamic instability requiring continuous intravenous medication or mechanical circulatory support treatment
∙ Echocardiographic estimation of PASP >70 mmHg or right heart catheterization measurement of PVR >3 Wood units
∙ Symptoms, signs, or echocardiographic evidence of severe right heart failure, such as TAPSE <15 mm or peak S-wave velocity <10 cm/s
∙ Severe liver cirrhosis with esophageal varices
∙ History of heart transplantation
∙ Severe hematological disorders
∙ Intracardiac mass or thrombus diagnosed by echocardiography
∙ Contraindications or high risk for TEE
∙ Poor quality of TEE imaging
∙ Known allergy or contraindication to medical agents used during the procedure
∙ Pregnancy or intended pregnancy within 12 months
∙ Anticipated need for emergency surgery or any elective cardiac surgery for any reason within 12 months,
∙ Non-cardiac disease resulting in a life expectancy of less than 12 months

CABG, coronary artery bypass grafting; DMR, degenerative mitral regurgitation; ECL, echocardiographic core laboratory; FMR, functional mitral regurgitation; HF, heart failure; LVESD, left ventricular end-systolic diameter; MR, mitral regurgitation; NYHA, New York Heart Association; PASP, pulmonary artery systolic pressure; PCI, percutaneous coronary intervention; PVR, pulmonary vascular resistance; S-wave,  systolic wave; TAPSE, tricuspid annular plane systolic excursion; TAVR, transcatheter aortic valve replacement; TEE, transesophageal echocardiography.

### 2.6 Subject Screening, Enrollment, and Follow-Up

#### 2.6.1 Screening

Eligible subjects for the TEER-CD trial are identified through a rigorous screening process that begins with a review of medical history and current clinical presentation. All potential participants must be aged 18 or older and diagnosed with symptomatic, moderate-to-severe (3+) or severe (4+) native commissural DMR. The diagnosis must be confirmed by an independent ECL using standardized assessments prior to enrollment. Patients are screened for the presence of specific morphological features characteristic of commissural DMR, including prolapse or flail involving the mitral valve commissures.

#### 2.6.2 Enrollment

Once potential participants have been identified and have met all inclusion criteria, they are provided with detailed information about the study, including the purpose, procedures, potential risks, and benefits. Informed consent is obtained from each participant, ensuring they understand the study requirements and their rights as research subjects.

#### 2.6.3 Follow-Up

After enrollment, participants undergo a comprehensive baseline assessment, which includes clinical examinations, echocardiography, and quality-of-life assessments. Following the M-TEER procedure, participants are closely monitored for the primary and secondary endpoints at predefined intervals: 30 days, 6 months, and 1 year post-procedure.

**Fig. 4.**
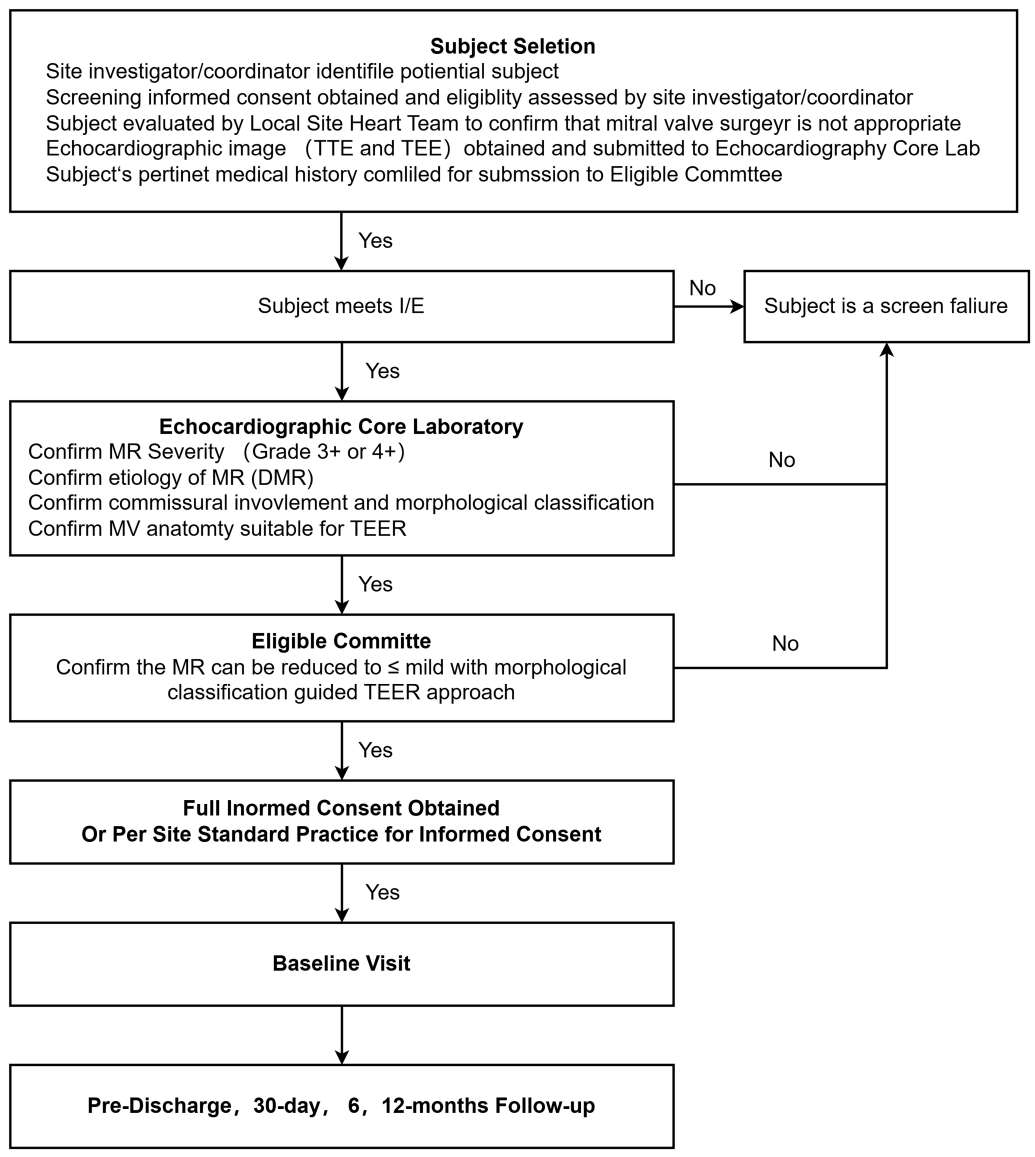
**Patient flow for screening, enrollment and follow up in the TEER-CD trial**. DMR,  degenerative mitral regurgitation; I/E,  inclusion and exclusion criteria; MR,  mitral regurgitation; TEE,  transesophageal echocardiography; TEER,  transcatheter edge-to-edge repair; TTE,  transthoracic echocardiography.

The 30-day follow-up focuses on early safety and procedural outcomes, while the 6-month and 1-year assessments evaluate the durability of the intervention and long-term safety. Each follow-up visit includes a physical examination, echocardiographic assessment, and evaluation of quality-of-life measures. Adverse events are captured and reported throughout the study period (Fig. 4).

In summary, the subject screening, enrollment, and follow-up processes in the TEER-CD trial are designed to be comprehensive, rigorous, and participant-centered, ensuring that the study generates high-quality data to evaluate the safety and effectiveness of the novel morphological classification-guided M-TEER approach for commissural DMR.

### 2.7 Study Endpoints

The comprehensive study endpoints are delineated in Table 2, with the principal study endpoints defined in accordance with the recommendations of MVARC [11]. The primary efficacy endpoint is the proportion of subjects with mild or less MR (≤1+), without mitral valve replacement, and without recurrent mitral valve intervention (surgical or percutaneous) from the time of the index procedure through 12  months. This endpoint is powered to demonstrate statistical significance and is intended to assess the effectiveness of the novel morphological classification-guided M-TEER strategy in reducing MR in patients with commissural DMR.

**Table 2.** **Study endpoints**.

Primary efficacy endpoint
∙ Proportion of subjects with mild or less MR (≤1+), without MV replacement, and without recurrent MV intervention (surgical or percutaneous) from the time of index procedure through 12 months
Secondary efficacy endpoints
∙ The success rate of TEER and the success rate of device implantation
■ The success rate of TEER is defined as no operative death, no surgical treatment, or reintervention related to device implantation or vascular access.
■ The success rate of device implantation is defined as successful device implantation 30 days after TEER, no MR (≥2+), no TEER-related mitral stenosis (mitral valve gradient ≥5 mmHg or effective regurgitant orifice area <1.5 cm^2^), no death and stroke, and no nonelective cardiac surgery due to device-related complications.
∙ Proportion of surviving patients with mild or less MR (≤1+) at 30 days and 6 months post TEER
∙ Proportion of surviving patients with moderate or less MR (≤2+) at 30 days, 6 months, and 12 months post TEER
∙ Hierarchical composite endpoint of death and recurrent HF hospitalization within 12 months post TEER
∙ Recurrent HF hospitalization within 12 months post TEER
∙ All-cause mortality within 12 months post TEER
∙ Improvement of KCCQ compared with baseline at 12 months
∙ Improvement of 6-minute walking distance from baseline at 12 months
∙ Proportion of NYHA functional class I or II subjects at 30 days, 6 months, and 12 months post TEER
∙ Change in LVEDV from baseline to 12 months
Secondary safety endpoints
∙ Composite endpoint of all-cause mortality, stroke, myocardial infarction, cardiac hospitalization, or nonelective cardiovascular surgery for device-related complications at 12 months
∙ Composite end point at 12 months of
-SLDA
-Device embolization
-Endocarditis requiring surgery
-ECL-confirmed mitral stenosis requiring surgery
-LVAD implant
-Heart transplant
-Any device-related complication requiring nonelective cardiovascular surgery
∙ Incidence of mitral chordae tendineae entanglement during TEER

ECL,  echocardiographic core laboratory; HF,  heart failure; KCCQ ,  Kansas City Cardiomyopathy Questionnaire; LVAD,  left ventricular assist device; LVEDV, left ventricular end-diastolic volume; MR,  mitral regurgitation; MV,  mitral valve; NYHA,  New York Heart Association; SLDA ,  single leaflet device attachment; TEER, transcatheter edge-to-edge repair.

Secondary efficacy endpoints encompass both procedural and clinical outcomes. Procedural outcomes include technical success and device success. Clinical outcomes include the proportion of surviving patients with MR  ≤1+ at 30 days and 6 months, MR  ≤2+ at 30 days, 6 months, and 12 months; the hierarchical composite of all-cause mortality and recurrent HF hospitalization within 12 months; recurrent HF hospitalization and all-cause mortality within 12 months; change in Kansas City Cardiomyopathy Questionnaire (KCCQ) score and 6-minute walk distance from baseline to 12 months; the proportion of patients in NYHA functional class I or II at 30 days, 6 months, and 12 months; and change in left ventricular end-diastolic volume (LVEDV) from baseline to 12 months.

Secondary safety endpoints include a composite of all-cause mortality, stroke, myocardial infarction, cardiac hospitalization, or nonelective cardiovascular surgery for device-related complications at 12 months. Additional safety endpoints comprise a composite at 12 months of single leaflet device attachment (SLDA), device embolization, endocarditis requiring surgery, ECL-confirmed mitral stenosis requiring surgery, left ventricular assist device (LVAD) implantation, heart transplantation, or any device-related complication requiring nonelective cardiovascular surgery. The incidence of mitral chordae tendineae entanglement during M-TEER will also be recorded.

### 2.8 Data Collection

Data collection is standardized across all participating centers using a centralized electronic data capture system. This system ensures the integrity and consistency of the data collected, facilitating accurate and timely analysis. All data are monitored by an independent data management committee, which oversees data quality and ensures adherence to the study protocol (**Supplementary Table 1**).

All required data for the trial will be collected on standardized Case Report Forms. All protocol-mandated echocardiograms and electrocardiograms will be sent to the ECL (Beijing Anzhen Hospital, China). Data management and study analyses will be performed by National Clinical Research Center for Cardiovascular Diseases (Beijing, China).

### 2.9 Statistical Considerations

#### 2.9.1 Sample Size Calculation

The primary endpoint of this study is the proportion of patients achieving a MR grade of ≤1+ without repeat mitral intervention at 1-year follow-up. The study is designed as a single-arm, objective performance goal (OPG) trial with the following statistical hypotheses:

*H₀: P_𝑇_ ≤ P₀*

*H₁: P_𝑇_ > P₀*

where *P*_𝑇_ represents the expected primary endpoint rate in this study, and *P₀* denotes the target performance level derived from previous literature.

Based on our center’s retrospective data and relevant published studies [16], the expected rate of the primary endpoint (*P*_𝑇_) was set at 85%. The target performance level (*P₀*) was set at 75%, reflecting clinically acceptable outcomes reported for complex MR populations in previous trials [17].

Using a one-sided significance level (α) of 0.025, a statistical power (1–β) of 80%, and assuming a 10% dropout rate at 12-month follow-up, the required sample size was calculated to be 148 patients. The sample size calculation was performed using the formula for a one-sample proportion test against a performance goal:

$$n=\frac{{\left[Z_{1-\alpha }\cdot \sqrt{P_{0}\,\left(1-P_{0}\right)}+Z_{1-\beta }\cdot \sqrt{P_{T}\,\left(1-P_{T}\right)}\right]}^{2}}{{\left(P_{T}-P_{0}\right)}^{2}}$$

where *Z*_1-α_ = 1.96 for a onesided α of 0.025, and *Z*_1-β_ = 0.84 for 80% power.

#### 2.9.2 Analysis Populations

2.9.2.1 Intention-to-Treat (ITT) Population

The ITT population includes all participants who provided informed consent and received the intervention. This population is used for the primary analysis of the intervention’s effectiveness, regardless of adherence to the study protocol or completion of the study.

2.9.2.2 Per Protocol (PP) Population

The PP population includes participants from the ITT population who adhered to the study protocol, completed all scheduled visits, and received the intervention as intended. This population is used for sensitivity analyses to assess the effectiveness of the intervention under optimal conditions.

In the TEER-CD trial, the primary efficacy endpoint will be rigorously assessed utilizing the ITT population. This approach ensures an unbiased evaluation of the novel morphological classification-guided M-TEER intervention, encompassing all participants who underwent the intervention as per the study design. The PP population will serve as the basis for sensitivity analyses, designed to scrutinize the durability of the treatment effect within a subgroup that strictly adhered to the study protocol. This methodological framework facilitates a comprehensive assessment of the intervention’s efficacy under both optimal (PP) and pragmatic (ITT) conditions, thereby offering a nuanced understanding of the intervention’s therapeutic potential. Concurrently, the safety population, which includes all individuals who received the intervention and had post-procedure safety assessments, will be employed to evaluate the safety endpoints.

#### 2.9.3 Statistical Analysis

Baseline characteristics, procedural data, and follow-up outcomes will be summarized using descriptive statistics. Continuous variables will be presented as means ± standard deviations (SD) if normally distributed, or as medians with interquartile ranges (IQR) if non-normally distributed. Categorical variables will be expressed as frequencies and percentages.

The primary efficacy analysis will be conducted in the ITT population using a one-sample proportion Z-test to evaluate whether the proportion of patients achieving MR ≤1+ at 12 months exceeds the prespecified OPG of 75%. A one-sided significance level of 0.025 will be applied to test the null hypothesis that the success rate is less than or equal to the OPG.

Secondary endpoints will be analyzed descriptively. Comparisons of continuous variables between baseline and follow-up will use paired *t*-tests or Wilcoxon signed-rank tests based on data distribution. Categorical variables will be compared using McNemar’s test or Cochran’s Q test, as appropriate. Time-to-event outcomes will be analyzed using Kaplan–Meier estimates, with median survival times and event-free rates calculated at 30 days, 6 months, and 12 months. Log-rank tests may be applied for subgroup comparisons where relevant.

To assess long-term durability, all subjects will be followed for up to five years. A secondary Bayesian analysis will be performed to estimate the posterior probability of sustained therapeutic success (defined as MR ≤1+ without mitral valve replacement or reintervention). This approach enables dynamic updating of treatment effect estimates over time and offers further insight into outcomes beyond the 12-month endpoint.

Predefined subgroup analyses will be performed according to the three morphological subtypes to evaluate the efficacy and safety of the classification-guided M-TEER strategy. Due to distinct procedural approaches and expected small sample sizes within subgroups, a dual analytic strategy will be employed: exact tests will be used for small-sample inference, while Bayesian methods will provide probabilistic estimations with credible intervals, incorporating prior knowledge to enhance robustness.

Sensitivity analyses will be conducted to evaluate the stability of the primary outcome results. These will include analyses based on the PP population—excluding major protocol deviations—and scenario-based imputations for missing data (e.g., worst-case and best-case assumptions). Consistency between the ITT and PP populations, as well as across imputation methods, will strengthen the reliability of trial conclusions.

All statistical analyses will be conducted using a one-sided alpha level of 0.025. Analyses will be performed using SAS software (version 9.4; SAS Institute Inc., Cary, NC, USA), R software (version 4.2.1; R Foundation for Statistical Computing, Vienna, Austria), and SPSS software (version 24.0; IBM Corp., Armonk, NY, USA).

## 3. Discussion

The TEER-CD trial represents a significant advancement in the field of interventional cardiology, addressing a critical gap in the management of commissural DMR. Utilizing a prospective, multicenter, single-arm, OPG design, the study adopts a refined TEER approach tailored to the unique anatomical challenges of commissural DMR. The implications of this research are far-reaching, as it could lead to a paradigm shift in the management of commissural DMR. With an aging global population and an increasing prevalence of valvular heart disease, the need for effective and less invasive treatment options is more critical than ever. The TEER-CD study is poised to make a significant contribution to the field, offering hope to patients who have historically been underserved by existing therapies.

### 3.1 Innovative Morphological Classification System

The introduction of a detailed morphological classification system is a cornerstone of this trial. By categorizing patients into distinct subtypes based on their echocardiographic features, the study allows for a personalized treatment strategy, optimizing the procedural approach for each patient’s unique valve morphology. This systematic categorization is expected to enhance procedural precision and outcomes, potentially reducing complications and improving repair efficacy.

### 3.2 Clinical Implications and Potential Impact

The implications of the TEER-CD trial are profound, promising to expand the applicability of M-TEER to a patient population that has historically been underserved by traditional methods. The study’s focus on commissural DMR is particularly noteworthy, given that this subset of patients often presents with more advanced disease at diagnosis and has been frequently excluded from clinical trials. The potential for this study to refine treatment guidelines and broaden the therapeutic scope of M-TEER is substantial.

### 3.3 Methodological Considerations

The use of an OPG design allows for targeted evaluation of the efficacy of the novel morphology classification-guided M-TEER strategy in patients with commissural DMR. This design is appropriate in the absence of an established comparator group for this anatomical subset. The total sample size of 148 patients is sufficient to support the primary efficacy objective and allows for descriptive and exploratory analyses of secondary endpoints. A planned 5-year follow-up, supplemented by a secondary Bayesian analysis at extended time points, will provide insights into the long-term durability of treatment effects beyond the 12-month primary analysis. To evaluate treatment effects across different anatomical subtypes, pre-specified subgroup analyses will be conducted. Given the relatively small sample sizes in each subgroup, the use of exact tests and Bayesian methods enhances the analytical robustness by enabling reliable inference without strict distributional assumptions and by incorporating prior information to refine estimates.

### 3.4 Limitations and Future Research

While the TEER-CD trial is methodologically robust, it is not without limitations. The single-arm design, though appropriate for this innovative intervention, limits the ability to make direct comparisons with other treatments. Future research should consider randomized controlled trials to directly compare the novel morphological classification-guided M-TEER with existing therapies.

Additionally, the generalizability of the study’s findings may be limited by the demographic and geographic diversity of the study population. Future studies should aim to include a more diverse patient population to enhance the global applicability of the results.

## 4. Conclusions

In summary, the TEER-CD trial is poised to make a significant contribution to the field of structural heart disease, particularly in the treatment of commissural DMR. By introducing a novel classification system and personalized procedural strategies, this study has the potential to transform clinical practice, offering new hope to patients with this complex and challenging condition. As the field of interventional cardiology continues to evolve, studies like TEER-CD are essential for driving innovation and improving patient outcomes.

## Data Availability

No datasets were generated or analyzed during the current study.

## Abbreviations

2D, two-dimensional; 3D, three-dimensional; AC, anterolateral commissure; ACC, American College of Cardiology; AHA, American Heart Association; Ant, anterior; AV, aortic valve; AVP, Amplatzer Vascular Plug; CABG, coronary artery bypass grafting; CD, commissural disease; DMR, degenerative mitral regurgitation; ECL, echocardiographic core laboratory; ESC, European Society of Cardiology; FMR, functional mitral regurgitation; HF, heart failure; IBM, International Business Machines Corporation; I/E, inclusion and exclusion criteria; IQR, interquartile range; ITT, intention-to-treat; KCCQ, Kansas City Cardiomyopathy Questionnaire; Lat, lateral; LAX, long-axis; LVAD, left ventricular assist device; LVEDV, left ventricular end-diastolic volume; LVESD, left ventricular end-systolic diameter; M-TEER, mitral transcatheter edge-to-edge repair; MC, mitral commissure; ME, mid-esophageal; Med, medial; MR, mitral regurgitation; MV, mitral valve; MVARC, Mitral Valve Academic Research Consortium; NYHA, New York Heart Association; OPG, objective performance goal; PASP, pulmonary artery systolic pressure; PC, posteromedial commissure; PCI, percutaneous coronary intervention; Post, posterior; PP, per-protocol; PVR, pulmonary vascular resistance; SAS, Statistical Analysis System; SAX, short-axis; SD, standard deviation; SLDA, single leaflet device attachment; SPSS, Statistical Package for the Social Sciences; TAPSE, tricuspid annular plane systolic excursion; TAVR, transcatheter aortic valve replacement; TEE, transesophageal echocardiography; TEER, transcatheter edge-to-edge repair; TTE, transthoracic echocardiography; USA, United States of America.

## Declaration of AI and AI-Assisted Technologies in the Writing Process

All authors confirm that they are fully aware of the use of AI-assisted technologies during the preparation of this work. Specifically, ChatGPT-4.0 was used solely for the purpose of language editing, such as spelling and grammar checking. The AI tool was not used for content generation or data analysis. All authors have thoroughly reviewed and verified the AI-assisted outputs to ensure the accuracy and integrity of the manuscript. The authors take full responsibility for the content and conclusions presented in this work.

## Supplementary Material

Supplementary material associated with this article can be found, in the online version, at https://doi.org/10.31083/RCM39373.
