# Supplementary material for: Safety and Efficacy of Novel Morphology Classification-Guided Mitral Valve Transcatheter Edge-to-Edge Repair for Patients With Commissural Degenerative Mitral Regurgitation: Design and Rationale of the TEER-CD Trial
Source: Rev Cardiovasc Med. 2025 Dec 19;26(12):39373. doi: 10.31083/RCM39373 (PMC12781021; doi:10.31083/RCM39373)
Supplement: Supplementary file 1 [file 2153-8174-26-12-39373-s1.docx]

**Supplemental Materials**


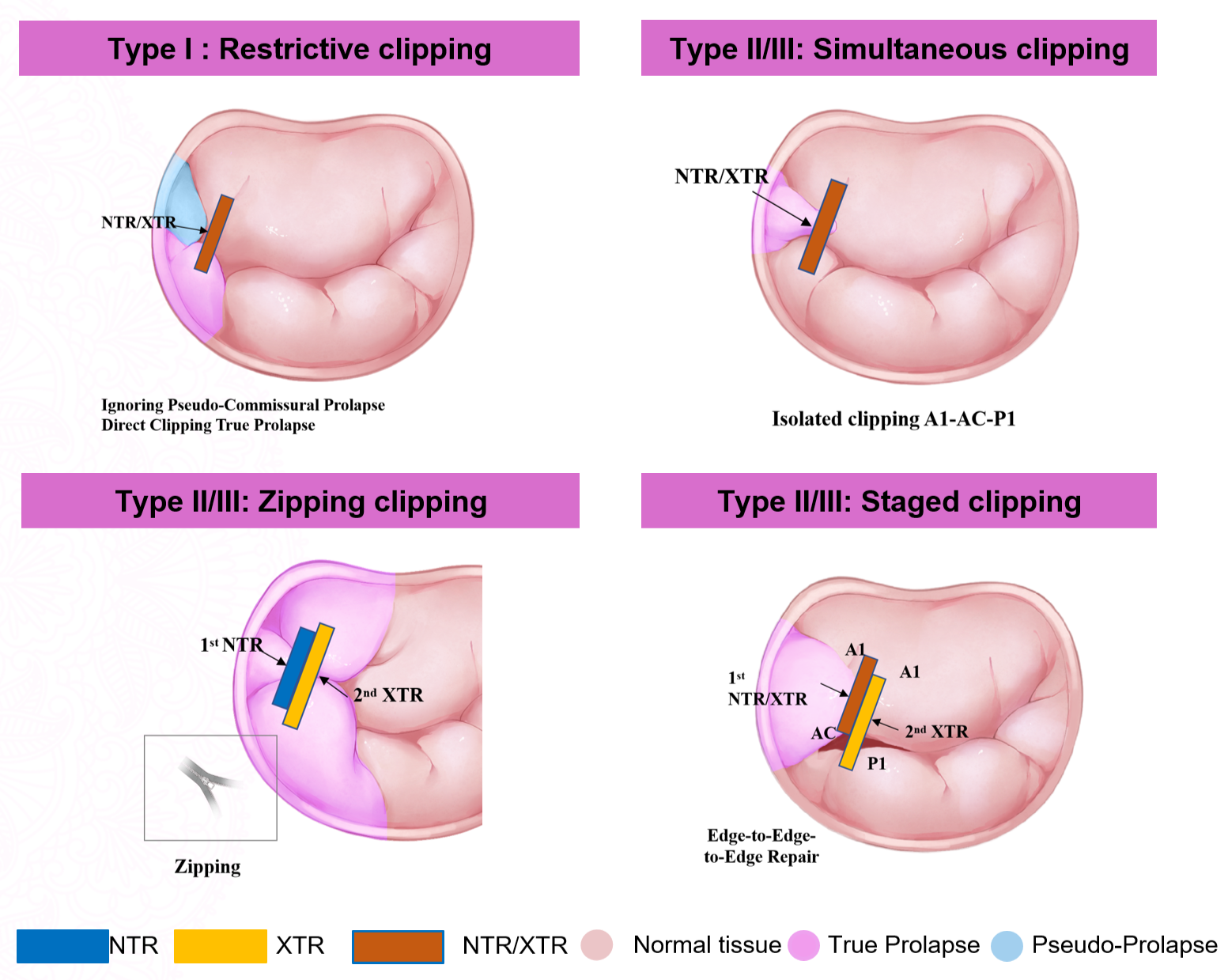


**Supplementary Fig. 1. Illustrative diagram of the morphology classification-guided M-TEER strategies for commissural DMR**

For Type I patients, a restrictive clipping strategy is planned, aiming to clip the true prolapsed lesion adjacent to the involved commissural area while restricting excessive motion of the commissural leaflet. For Type II and III patients, leaflet length and commissural space are assessed to determine the feasibility of simultaneously grasping the three coaptation lines (A-[C]-P). If feasible, a simultaneous clipping strategy is applied, grasping A-[C]-P in a single attempt; persistent significant regurgitation after the first clip warrants additional clips for the remaining diseased segments (“zipping” strategy). If simultaneous grasping is not possible, a staged clipping strategy is recommended: the first clip grasps A-C or P-C to create a tissue bridge, followed by a second clip to complete repair with the adjacent leaflet.

| **Supplementary Table 1. Study Data Collection** | | | | | | |
| --- | --- | --- | --- | --- | --- | --- |
| **Data collection** | **Baseline** | **D1** | **D7/at discharge** | **D30** | **D180** | **D365** |
|  | **-7 Days** | **0 D** | **0 D** | **± 7d** | **± 7d** | **± 30d** |
| Inform consent | X |  |  |  |  |  |
| Demography | X | X |  |  |  |  |
| Past history | X |  |  |  |  |  |
| Vital sign |  | X | X | X | X | X |
| Physical Examination |  | X | X | X | X | X |
| History of angina |  | X | X | X | X | X |
| NYHA Functional Class |  | X | X | X | X | X |
| STS score |  | X |  |  |  |  |
| Modified Rankin Scale (MRS) score |  | X | X | X | X | X |
| KCCQ score |  | X | X |  |  |  |
| 6-min walk distance |  | X | X |  |  |  |
| ECG (18 leads) |  | X | X | X | X | X |
| TTE |  | X | X | X | X | X |
| TEE | X |  |  |  |  |  |
| CBC |  | X | X |  |  |  |
| NT-proBNP |  | X |  |  |  |  |
| Coagulation Test |  | X | X |  |  |  |
| Hepatic and renal function |  | X | X |  |  |  |
| TNI/TNT |  | X | X |  |  |  |
| Medication |  | X | X | X | X | X |
| Procedural characteristics* |  | X |  |  |  |  |
| Other treatments |  | X | X | X | X | X |
| AE/SAE Events |  | X | X | X | X | X |
| NYHA New York Heart Association, STS The Society of Thoracic Surgeon, KCCQ Kansas City Cardiomyopathy Questionnaire, ECG electrocardiography, TTE transthoracic echocardiography, TEE transesophageal echocardiography, CBC complete blood counts NT-proBNP N-terminal fragment brain natriuretic peptides, TNT/TNI troponins T and I, AE/SAE adverse events/severe adverse events  Procedural characteristics include commissural prolapse morphological classification, device type, device number, device location, procedural time, fluoroscope time, procedural success rate, technical sussess rated, device-related complication and AE/SAE according to the MVARC criteria. | | | | | | |
